# Opening the door to multi-year marine habitat forecasts

**DOI:** 10.1038/s41467-024-45020-9

**Published:** 2024-01-31

**Authors:** Mark R. Payne

**Affiliations:** National Center for Climate Research (NCKF), Danish Meteorological Institute (DMI), 2100 Copenhagen, Denmark

**Keywords:** Climate-change ecology, Projection and prediction

## Abstract

Combining ocean predictions with physiological understanding yields the ability to forecast habitat multiple years into the future for a wide variety of marine organisms. However, several challenges remain before we see the regular production and use of marine habitat forecasts.

A well-known quote, often attributed to the Danish physicist Niels Bohr, has it that it is difficult to make predictions, particularly if they are about the future. Bohr’s assertion is however contradicted every day by reliable weather forecasts that inform our daily routine and choice of activities. But what if we could see even further into the future, for example, months, years or even a decade ahead? How would that change the way that we plan and behave? Chen et al.^1^ open the door to this tantalising possibility by providing a general demonstration of the ability to predict the habitat of marine organisms on a multi-year timescale. This result throws open the door to new forecast applications and new approaches to the management of living marine resources.

Chen et al.’s new result builds in the first place on the recent development of skilful multi-year climate prediction systems^2^. Climate prediction can be best understood as the application of climate models in a manner similar to a modern weather forecast system, but with the focus being on characterising the statistical properties of the weather months and years into the future (rather than forecasting the actual weather on a given day). High-prediction skill is particularly apparent in the ocean, where the slower dynamics and high inertia (compared to the atmosphere) enable long prediction horizons. Forecast skill on the decadal scale has been demonstrated for many aspects of the ocean, including sea surface temperature, heat content, and ocean circulation^3–5^.

The ability to predict the state of the ocean is of particular relevance to the management of living marine resources, as these organisms are strongly influenced by variations in conditions in the ocean. Most marine organisms are “cold-blooded” and their body temperature (and thus physiology) is at the same temperature as the surrounding water: variations in water temperature therefore have a direct impact on the functioning of the organism. Such variations manifest themselves in many different ways, including impacts on reproduction, the timing of key events such as migration and spawning, and where species are found (their distribution). For the human industries that depend upon living marine resources, this variability can pose tremendous challenges, and the ability to foresee changes in the future therefore has the potential to improve both productivity and sustainability^6^. In a few select cases, it has been possible to develop short-term marine ecological forecast products^7^ to support decision making, particularly with regards to the distribution of species.

Chen et al. consider the problem of how to take advantage of decadal predictability of the state of the ocean (e.g. temperature, salinity, etc) to forecast biologically relevant metrics. While they are not the first to do so, previous efforts have been focussed on individual species^8^ using established understanding of how each is influenced by the environment. While such an approach is quite valid, the highly specific nature of the analysis makes it difficult to apply to other species.

Chen et al. however, take a uniquely generic approach to the problem by considering the physiological constraints on the habitat of a species. In particular, they apply a generic metabolic index^9^, based on a physiological model of an organism’s tolerance for low-oxygen conditions, and how this tolerance varies with temperature. The response of species as diverse as sea squirts (Fig. 1), crabs and shrimp can then be characterised in terms of two simple parameters. Predictions of temperature, oxygen, and salinity derived from climate prediction systems can then be used to generate predictions of the metabolic index.

**Fig. 1:**
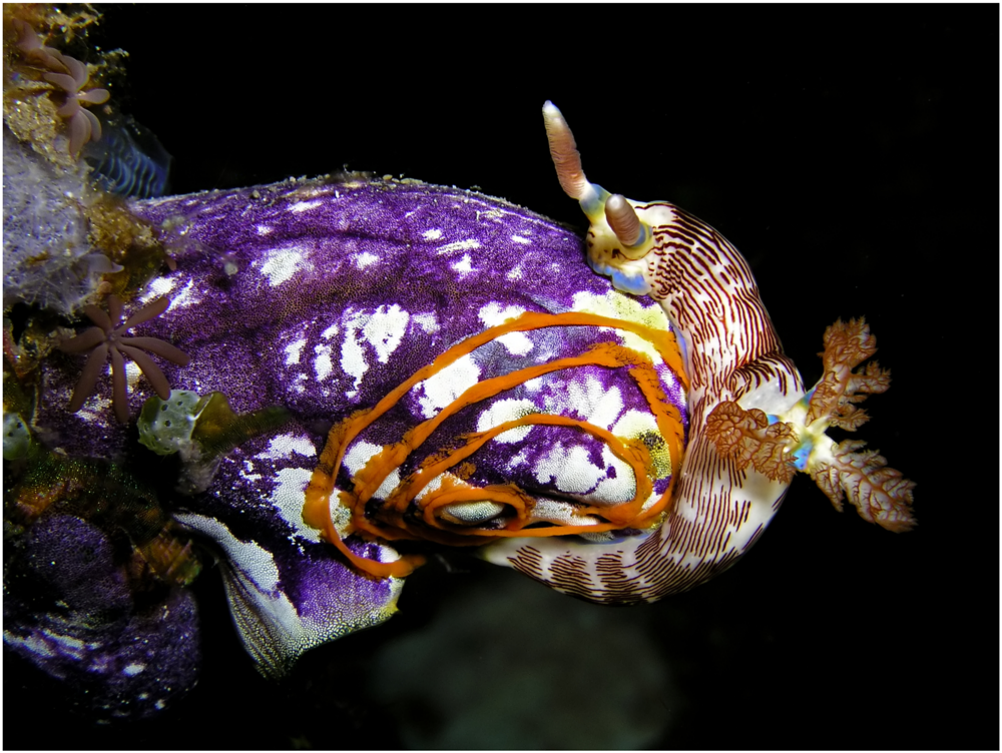
A nudibranch (*Nembrotha lineolata*) lays an egg spiral on a sea squirt (*Polycarpa aurata*) near Metinaro, East Timor. Chen et al. show the ability to forecast habitat suitability of species, such as the sea squirt, multiple years into the future. Photo credit: “Seasquirt”, by Nick Hobgood (Own work) [CC-BY-SA-3.0 https://creativecommons.org/licenses/by-sa/3.0/] via Wikimedia Commons. Available at https://commons.wikimedia.org/wiki/File:Seasquirt.jpg.

Chen et al. show that their forecasts skilfully predict the metabolic index multiple years into the future. They are able to decompose this predictability into its various source components (e.g. temperature, salinity, oxygen) and show that it is dominated by the oxygen component. Most importantly, they also examine the ability to make predictions across the full range of organismal traits considered, showing the presence of multiyear predictability for most combinations of factors. Chen et al. therefore demonstrate multiyear predictability of species habitat to be the rule, rather than the exception.

While Chen et al.’s work firmly establishes the scientific basis for habitat prediction, many challenges remain before marine ecological forecast products based on this work can start to appear. In particular, the ability to predict habitat, as demonstrated here, is not necessarily the same as the ability to predict the distribution of a species. Habitat is the potential area where a species can live, whereas the realised distribution (where species actually are found) is only a subset of the total available habitat. Many other factors beyond metabolic constraints influence distribution, including the availability of food and prey, the presence of predators and behavioural processes such as schooling dynamics and the need to reproduce. The ability to predict habitat does not therefore automatically confer the ability to predict the distribution of a species. Nevertheless, the presence of suitable habitat is a necessary condition for a species to be present in a given location, and can therefore provide the mechanistic basis for predictions of distribution, particularly in regions where distribution is constrained by habitat availability (e.g. on the edges of the distribution).

How such habitat and/or distribution forecasts can be used in a decision making context is also an outstanding issue. The management of both living marine resources and the industries that depend on them are very commonly focused on the next year and there are currently few existing applications where such multi-year forecasts can potentially contribute. Furthermore, the available habitat and distribution of a species (as studied here) is of lesser importance to fisheries management compared to the dominating question about the productivity (and therefore the fishing quota) in coming years. Exploiting the potential of these forecasts requires a dedicated focus on how they can be applied in a marine management context.

This work also highlights the need for better observations of oxygen in the ocean. The habitat predictability demonstrated by Chen et al. is dominated by the oxygen component of the metabolic index. However, oxygen observations in the ocean are relatively limited in scope: Chen et al. for example were forced to use a reconstruction of ocean oxygen, rather than the type of direct observations available for e.g. temperature, which can be measured remotely from satellite. An increased focus on ocean oxygen measurements can therefore potentially benefit our ability to predict ecologically relevant metrics, such as habitat, and indeed may be a prerequisite for producing ecological forecasts operationally.

In conclusion, Chen et al.’s work throws the door to multi-year ecological prediction wide open. Whereas predictability has previously been demonstrated in single cases, these new results show that habitat can be predicted generally across the full spectrum of species. While it still may be hard to make predictions, particularly about the future, the scientific basis for habitat forecasts multiple years ahead now appears to be in place.
